# Anti-PD-1/PD-L1 antibody therapy for pretreated advanced nonsmall-cell lung cancer: A meta-analysis of randomized clinical trials

**DOI:** 10.1097/MD.0000000000004611

**Published:** 2016-09-02

**Authors:** Guo-Wu Zhou, Ye Xiong, Si Chen, Fan Xia, Qiang Li, Jia Hu

**Affiliations:** a Department of Respiratory and Critical Care Medicine, Changhai Hospital, Second Military Medical University; b Department of Pulmonary Medicine, Medicine, 85 Hospital of People's Liberation Army, Shanghai; c Department of Oncology, The First Affiliated Hospital to PLA General Hospital, Beijing, P.R. China.

**Keywords:** docetaxel, nonsmall-cell lung cancer, PD-1, PD-L1

## Abstract

**Background:**

Anti-PD-1/PD-L1 antibody therapy is a promising clinical treatment for nonsmall-cell lung cancer (NSCLC). However, whether anti-PD-1/PD-L1 antibody therapy can provide added benefits for heavily pretreated patients with advanced NSCLC and whether the efficacy of anti-PD-1/PD-L1 antibody therapy relates to the tumor PD-L1 expression level remain controversial. Thus, this meta-analysis evaluated the efficacy and safety of anti-PD-1/PD-L1 antibody therapy for pretreated patients with advanced NSCLC.

**Methods:**

Randomized clinical trials were retrieved by searching the PubMed, EMBASE, ASCO meeting abstract, clinicaltrial.gov, and Cochrane library databases. The pooled hazard ratios (HRs) for overall survival (OS) and progression-free survival (PFS), and odds ratios for the overall response rate and adverse events (AEs) were calculated by STATA software.

**Results:**

Three randomized clinical trials involving 1141 pretreated patients with advanced NSCLC were included. These trials all compared the efficacy and safety of anti-PD-1/PD-L1 antibodies (nivolumab and MPDL3280A) with docetaxel. The results suggested that, for all patients, anti-PD-1/PD-L1 therapy could acquire a greater overall response (odds ratio = 1.50, 95% CI: 1.08–2.07, *P* = 0.015, *P* for heterogeneity [*P*_h_] = 0.620) and longer OS (HR = 0.71, 95% CI: 0.61–0.81, *P* < 0.001, *P*_h_ = 0.361) than docetaxel, but not PFS (HR = 0.83, 95% CI: 0.65–1.06, *P* = 0.134; *P*_h_ = 0.031). Subgroup analyses according to the tumor PD-L1 expression level showed that anti-PD-1/PD-L1 therapy could significantly improve both OS and PFS in patients with high expressions of PD-L1, but not in those with low expressions. Generally, the rates of grade 3 or 4 AEs of anti-PD-1/PD-L1 therapy were significantly lower than that of docetaxel. However, the risks of pneumonitis and hypothyroidism were significantly higher.

**Conclusion:**

Anti-PD-1/PD-L1 antibody therapy may significantly improve the outcomes for pretreated advanced NSCLC patients, with a better safety profile than docetaxel.

## 1 Introduction

Lung cancer is the leading cause of cancer-related death worldwide, with a 5-year overall survival (OS) rate of only 10% to 15%.^[1]^ Nonsmall-cell lung cancer (NSCLC), including adenocarcinoma and squamous cell carcinoma, comprises approximately 80% to 85% of all lung cancer cases. More than 60% of newly diagnosed NSCLC patients present with locally advanced or metastatic disease,^[2]^ which correlates closely with poor prognosis and high mortality.

Patients with advanced NSCLC whose disease progresses during or after first-line chemotherapy have limited options. Since the approval of docetaxel as second-line treatment in 1999, after it was shown to provide longer survival than best supportive care,^[3,4]^ little therapeutic progress has been made for squamous cell carcinoma, despite the benefits associated with docetaxel being modest. Although pemetrexed and erlotinib have better side-effect profiles than docetaxel for nonsquamous NSCLC, they have failed to show superiority to docetaxel concerning OS when used as second-line therapy.^[5,6]^

The programmed death 1 (PD-1) receptor, which is expressed on activated T cells, is activated by the tumor-expressed ligands PD-L1 and PD-L2. The interaction of PD-1 with PD-L1 and PD-L2, which are expressed prevalently in NSCLC, downregulates T cell activation and promotes tumor immune escape.^[7–9]^ Anti-PD-1/PD-L1 therapy uses PD-1/PD-L1 immune-checkpoint-inhibitor antibodies to disrupt PD-1/PD-L1-mediated signaling and restore antitumor immunity. Furthermore, anti-PD-1/PD-L1 therapy has been reported to be useful for the treatment of cancers with various types of histologic features.^[10–13]^

As the mechanism by which tumor cells escape recognition and elimination by the immune system is being revealed, many single arm studies concerning the efficacy of anti-PD-1/PD-L1 antibodies have been conducted; these have demonstrated that inhibition of the PD-L1/PD-1 pathway shows encouraging results on survival among all NSCLC subtypes.^[10,12,14]^ However, whether anti-PD-1/PD-L1 antibody therapy could provide added benefits for heavily pretreated patients with advanced NSCLC and whether the efficacy of this treatment relates to the tumor PD-L1 expression level remain unclear. To answer these questions, several randomized trials concerning the efficacy and toxicities of anti-PD-1/PD-L1 antibodies have been conducted.^[15–17]^ However, the results have been inconsistent and inconclusive, largely owing to the relatively small sample sizes of the individual studies. Thus, to better clarify these issues, we performed a meta-analysis on the efficacy and safety of anti-PD-1/PD-L1 antibody therapy for previously treated patients with advanced NSCLC.

## 2 Patients and methods

The current literature-based meta-analysis was performed to evaluate the efficacy and safety profile of anti-PD-1/PD-L1 antibodies for previously treated advanced NSCLC. All analyses were based on previous published studies, thus no ethical approval and patient consent are required.

### 2.1 Search strategy

A literature search of PubMed, EMBASE, ASCO meeting abstracts, clinicaltrial.gov, and Cochrane library (until November 11, 2015) was conducted using the following terms: “Carcinoma, Non Small-Cell Lung” [MeSH] or “NSCLC,” “PD-1” or “PD-L1,” and “Nivolumab” or “MPDL3280A” or “Pembrolizumab,” without restriction on language. The retrieved literatures were then read in their entirety to assess their appropriateness for the inclusion in this meta-analysis by 2 authors (GWZ and YX) independently. The reference lists of reviews and the retrieved articles were searched simultaneously to find additional eligible studies. If studies had partly overlapped subjects, the study with the larger sample size was selected. Any disagreement was resolved by discussion between the 2 authors.

### 2.2 Outcome for analysis

The efficacy outcomes analyzed were the OS, progression-free survival (PFS), and overall response rate (ORR). The safety outcomes analyzed were the adverse events (AEs), including fatigue, nausea, decreased appetite, diarrhea, anemia, neutropenia, pneumonitis, and hypothyroidism, among others.

### 2.3 Selection criteria

The included studies had to fulfill the following selection criteria: published, randomized clinical trials comparing anti-PD-1 or anti-PD-L1 therapy with chemotherapy; including patients with advanced or metastatic NSCLC after failure of previous treatments; and in which the outcomes were estimated by OS or PFS or ORR.

### 2.4 Qualitative assessment

The quality of the trials was assessed using the method reported by Jadad et al, which is based on the following 3 questions: whether an appropriate randomization method was reported (0–2 points); whether an appropriate blinding method was reported (0–2 points); and whether withdrawals and dropouts were reported (0–1 point). A trial with fewer than 3 points was considered as low-quality, while other trials (≥3 points) were considered as high-quality trials.^[18]^

### 2.5 Data extraction

The following variables were extracted from each study, if available: first author, year of publication, quality scores, comparison arms, number of patients in each arm, hazard ratios (HRs) for OS and PFS and their 95% confidence intervals (CIs), ORR, PD-L1 expression levels, and any grade and grade 3/4 AEs. All data were independently extracted by 2 investigators (GWZ and YX), who were blinded to the other author's findings, using a standardized data reporting form. Any disagreement between the 2 data extractors were resolved by consultation with 1 independent expert (HU).

The author-reported HRs with 95% CIs were used if possible. When 95% CIs were not directly reported in the original study, they were estimated indirectly using the *P*-value of the log-rank statistics.^[19]^

### 2.6 Statistical analysis

The pooled HRs with 95% CIs for OS and PFS, and odds ratios (ORs) with 95% CIs for ORR and AEs were calculated using the STATA SE 10.0 package (StataCorp, College Station, TX). HRs >1 favored the chemotherapy arm whereas HRs <1 favored the anti-PD-1/PD-L1 therapy arm. ORs for ORR and AEs >1 reflected a higher overall response and toxicity, respectively, in the immunotherapy arm. *P* values <0.05 were considered significant. Statistical heterogeneity among the trials was assessed using the standard *χ*^2^*Q* test and was considered statistically significant at *P* < 0.10. A fixed-effect model (the Mantel–Haenszel method) was used when heterogeneity was absent.^[20]^ Otherwise, a random-effect model (the DerSimonian and Laird method) was used.^[21]^ Subgroup analysis was performed according to the PD-L1 expression level. Potential publication bias was examined by funnel plots and Egger test,^[22]^ with *P* < 0.05 considered a significant publication bias.

## 3 Results

### 3.1 Characteristics of the included trials

**Figure 1:**
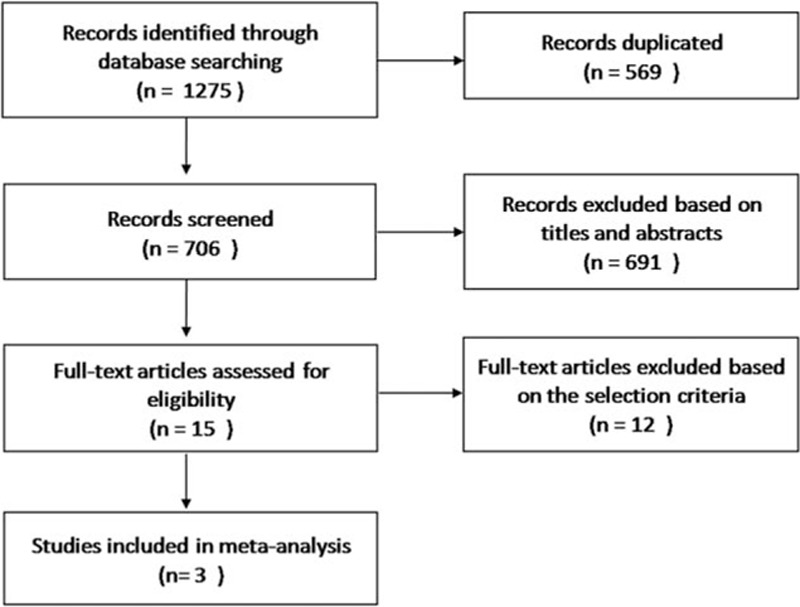
Flow diagram of the literature search and study selection process.

After a thorough electronic search, 1275 reports were identified; of these, 3 randomized trials involving 1141 pretreated patients with advanced NSCLC met the selection criteria and were included in the final analysis (Fig. 1). One of the included studies was an ASCO meeting abstract with available data. The main characteristics of the included trials are listed in Table 1. Among these trials, all of which were considered high-quality trials, 2 kinds of anti-PD-1/PD-L1 antibodies, MPDL3280A and nivolumab, were used in the immunotherapy arm, while only docetaxel was used in the chemotherapy arm. Subgroup analyses according to the PD-L1 expression were conducted in all these trials to explore the correlations between the PD-L1 expression level and immunotherapy efficacy.

**Table 1:**
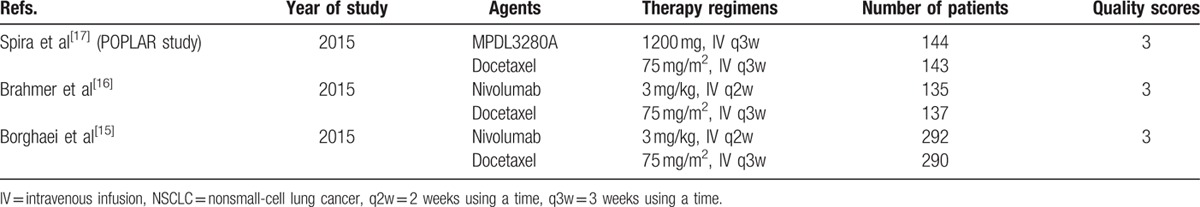
Characteristics of the 3 randomized controlled trials comparing anti-PD-1/anti-PD-L1 therapy with chemotherapy for previously treated advanced NSCLC.

### 3.2 Meta-analysis results of efficacy outcomes

**Figure 2:**
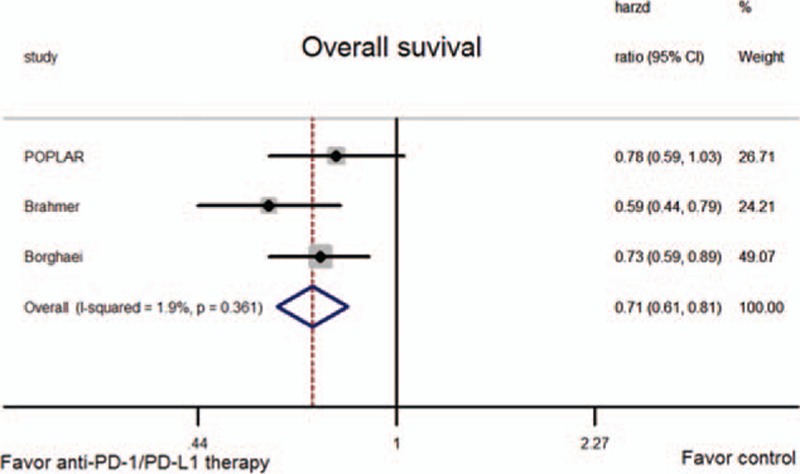
Meta-analysis of overall survival (OS).

**Figure 3:**
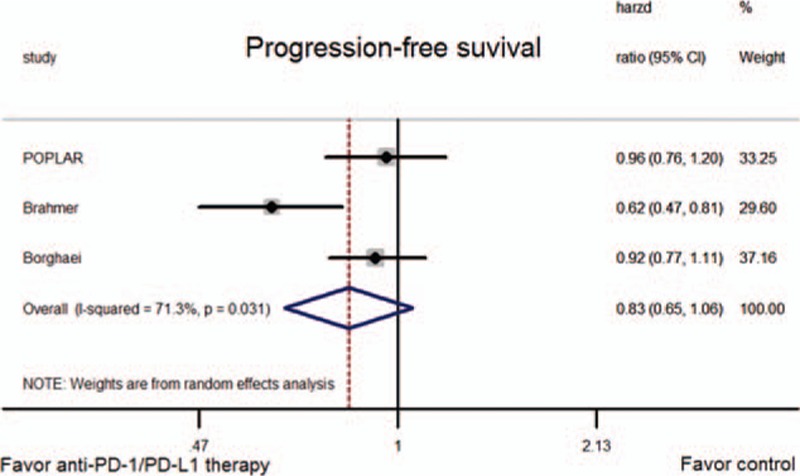
Meta-analysis of progression-free survival (PFS).

HRs for OS and PFS were available for all trials. The pooled HR showed a significant improvement in OS for anti-PD-1/PD-L1 therapy (HR = 0.71, 95% CI: 0.61–0.81, *P* < 0.001; *P*-value of heterogeneity [*P*_h_] = 0.361; Fig. 2), but not PFS (HR = 0.83, 95% CI: 0.65–1.06, *P* = 0.134; *P*_h_ = 0.031; Fig. 3).

**Figure 4:**
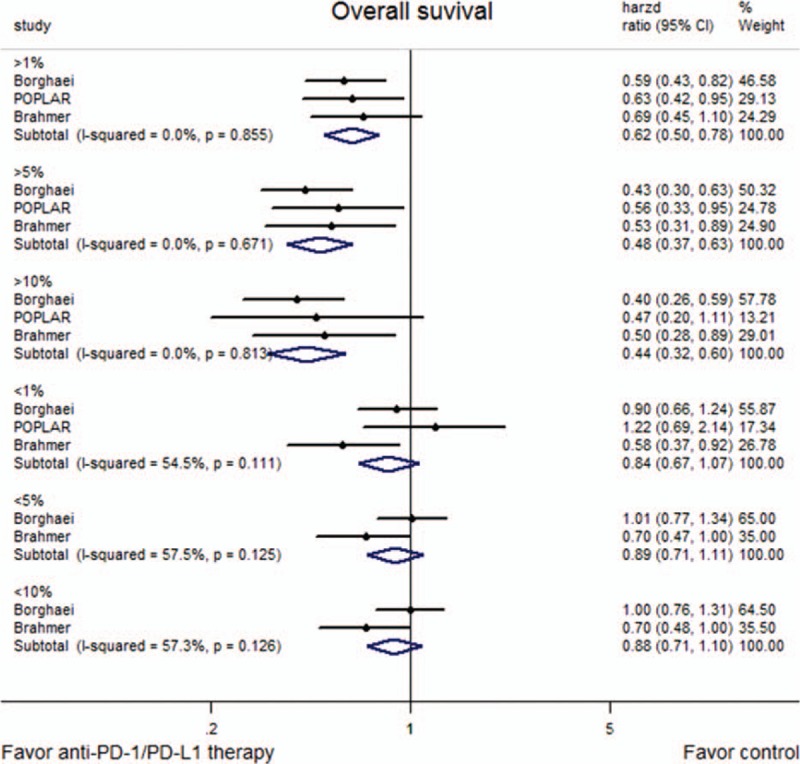
Forest plots describing the subgroup analyses of the associations between overall survival (OS) and programmed death-ligand 1 (PD-L1) expression at prespecified levels of 1%, 5%, and 10%.

**Figure 5:**
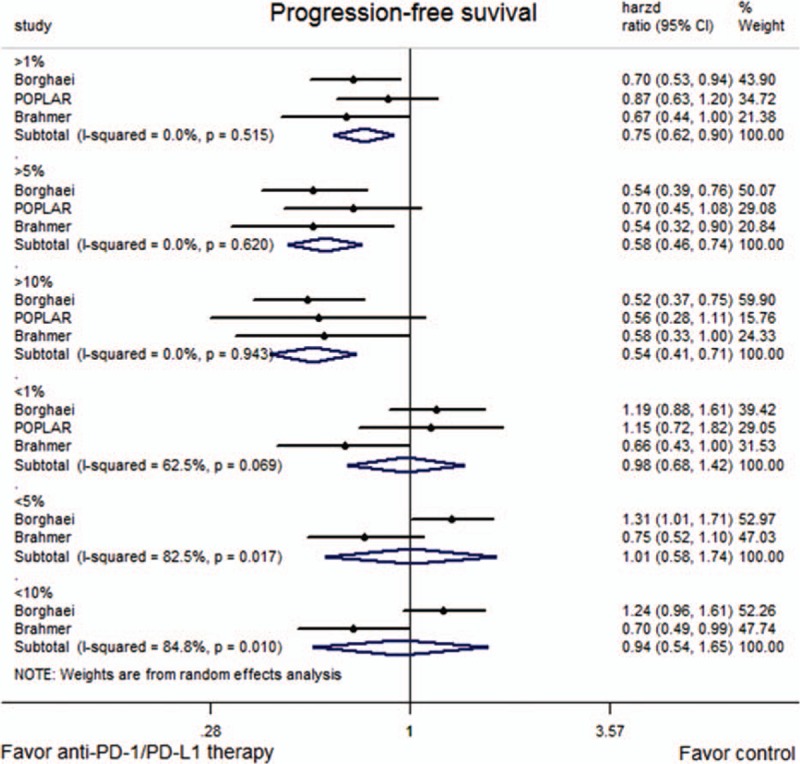
Forest plots describing the subgroup analyses of the associations between progression-free survival (PFS) and programmed death-ligand 1 (PD-L1) expression at prespecified levels of 1%, 5%, and 10%.

Subgroup analyses according to the tumor PD-L1 expression level showed that anti-PD-1/PD-L1 therapy significantly improved both OS (Fig. 4) and PFS (Fig. 5) in patients with high expressions of PD-L1, but not in those with low expressions. The results were similar irrespective of whether the PD-L1 expression was categorized as a level of 1%, 5%, or 10%.

**Figure 6:**
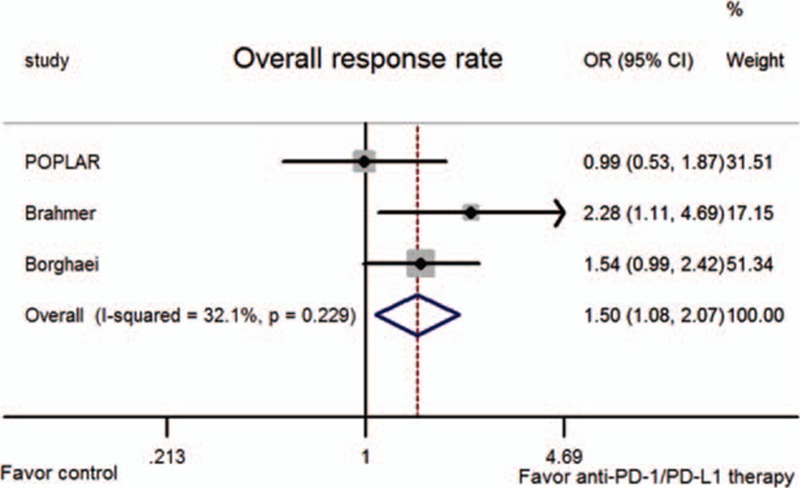
Meta-analysis of the overall response rate (ORR).

All trials reported the overall response in both arms. When the results of all trials were pooled, anti-PD-1/PD-L1 therapy was found to result in a greater overall response than docetaxel (OR = 1.50, 95% CI: 1.08–2.07, *P* = 0.015; *P*_h_ = 0.620; Fig. 6).

### 3.3 Meta-analysis results of safety outcomes

**Figure 7:**
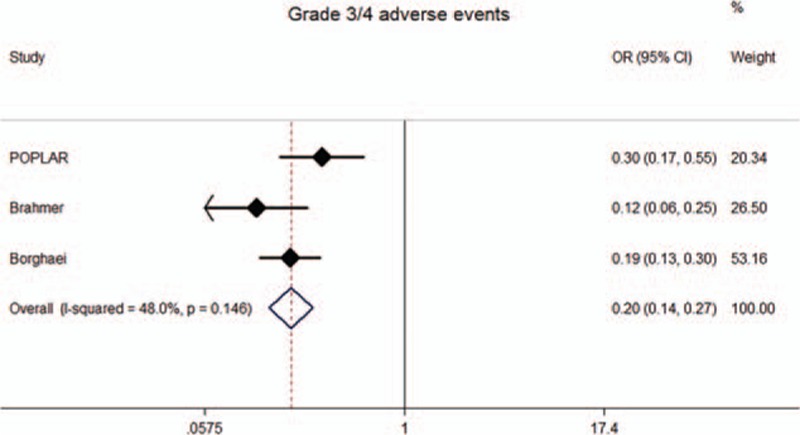
Meta-analysis of grade 3 or 4 adverse events (AEs).

**Figure 8:**
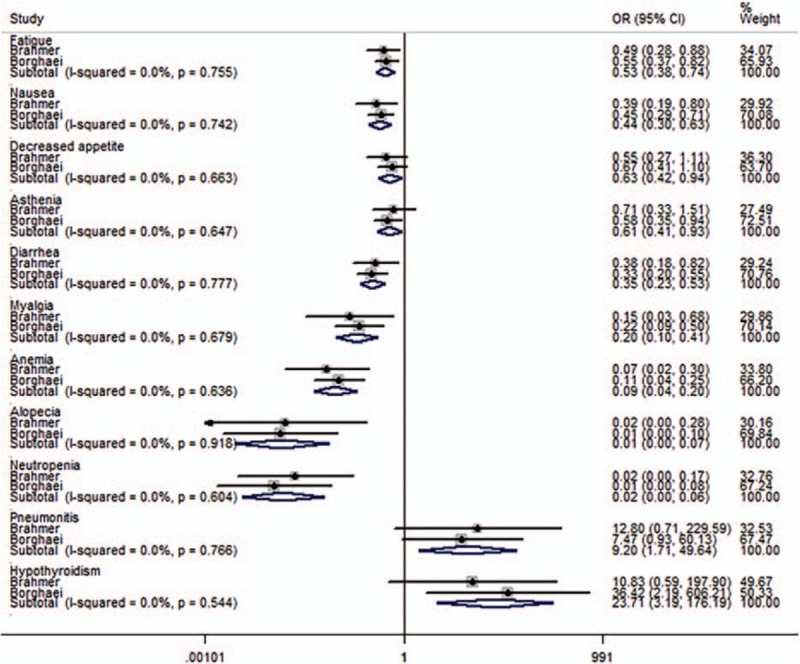
Meta-analysis of any grade adverse events (AEs).

All studies reported the grade 3 or 4 AEs, and 2 studies listed the items of specified events. Meta-analysis showed that the rates of grade 3 or 4 AEs of anti-PD-1/PD-L1 therapy were significantly lower than those of docetaxel (Fig. 7). For any grade AEs, the rates hematological AEs, such as anemia and neutropenia, and gastrointestinal reactions, such as nausea, decreased appetite, and diarrhea, were all significantly lower than in the docetaxel arm. However, the risks of pneumonitis and hypothyroidism were significant higher in the immunotherapy arm (Fig. 8).

### 3.4 Publication bias

**Figure 9:**
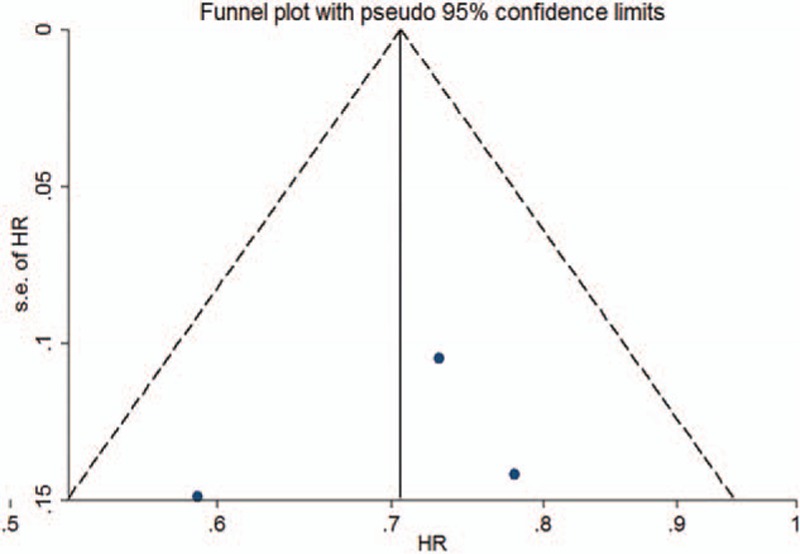
Funnel plot showing the publication bias of the included studies.

The funnel plot (Fig. 9) and Egger test (*P* = 0.715) indicated that no significant publication bias existed in this meta-analysis.

## 4 Discussion

The PD-1 receptor is expressed on activated T cells; when it binds to one of its key ligands, PD-L1 or PD-L2, T-cell activation is inhibited and the antitumor immune response is dampened. Many tumor cells, including NSCLC, overexpress PD-L1 to evade the immunological surveillance.^[23,24]^ Accordingly, several drugs targeting this pathway have been developed, including the anti-PD-1 antibodies nivolumab (BMS-936558), AMP-224, pembrolizumab (MK-3475), and pidilizumab (CT-011), and the anti-PD-L1 antibodies MPDL3280A, MEDI-4736, and BMS-936559 (MDX-1105). Some of these agents have been determined to be effective and safe in advanced NSCLC patients following prior chemotherapy. Moreover, several phase III trials^[15–17]^ have compared anti-PD-1/PD-L1 drugs with docetaxel in previously treated patients with advanced NSCLC. Although PD-1 and PD-L1 antibodies target different molecules in this pathway, clinical studies have demonstrated similar outcomes with both drugs. The phase III study by Borghaei et al^[15]^ showed that the median OS by treatment with nivolumab, an anti-PD-1 antibody, was 12.2 months, as compared with 9.4 months for treatment with docetaxel in previously treated advanced NSCLC patients. Another study by Spira et al^[17]^ showed that MPDL3280A, a PD-L1 antibody, resulted in a median OS of 11.4 months, as compared with 9.5 months for docetaxel. Their response rates were also similar (19% vs 15%). As a result, the current meta-analysis incorporating all available data from relative studies was deemed necessary to examine the current evidence.

This literature-based meta-analysis involving 1141 previously treated patients with advanced NSCLC who showed disease progression during or after first-line chemotherapy showed encouraging results; the findings indicated that anti-PD-1/PD-L1 antibody therapy could significantly improve the ORR and OS compared with single-agent docetaxel chemotherapy alone, without evidence of statistical heterogeneity.

Concerning the PFS, the results showed no significant effect of anti-PD-1/PD-L1 antibody therapy in the overall study population (*P* = 0.134). However, there was statistical heterogeneity between the 3 included trials, with the study by Brahmer et al^[16]^ showing that anti-PD-1/PD-L1 therapy could improve PFS compared to docetaxel, while the other 2 did not. Differences in the patient characteristics might contribute to the increased clinical heterogeneity. However, in order to prevent clinical heterogeneity induced by varying expression levels of PD-L1, subgroup analyses were performed according to the expression of PD-L1. In Brahmer et al's study, the percentages of patients whose tumors expressed PD-L1 at ≥1%, ≥5%, and ≥10% during immunotherapy were 54%, 36%, and 31%, respectively. In the POPLAR study and Borghaei et al's study,^[15,17]^ the corresponding proportions were 66%, 35%, and 17% and 53%, 41%, and 37%, respectively. The rates were similar between the immunotherapy and docetaxel groups. The results of the subgroup analyses showed that anti-PD-1/PD-L1 therapy significantly improved the PFS in patients with higher expression of PD-L1, but not in those with low expression, irrespective of the cut-off used. These results suggest that patients with overexpression of PD-L1, as a predictor of sensitivity to anti-PD-1/PD-L1 drugs, could achieve prolonged PFS from anti-PD-1/PD-L1 therapy. This also implies that a subset of patients would especially benefit from PD-1/PD-L1 blockers, and further exploration of this finding is needed. In terms of the OS, a meaningful separation of the OS curves was observed across all prespecified expression levels, consistent with the results of PFS, and there was a trend toward a longer OS as the PD-L1 expression level increased (PD-L1 expression ≥1%: HR = 0.62; ≥5%: HR = 0.48; ≥10%: HR = 0.44; Fig. 4).

The safety and toxicities of anti-PD-1/PD-L1 therapy were also explored in this meta-analysis. Anti-PD-1/PD-L1 therapy showed lower risks of grade 3 or 4 AEs than docetaxel. For any grade AEs, hematological AEs, such as anemia and neutropenia, and gastrointestinal reactions, such as nausea, decreased appetite, and diarrhea, were all significantly less common with anti-PD-1/PD-L1 therapy. We speculate that the reason for this finding is that docetaxel has many general properties of chemotherapy and can hence injure epithelium-derived cells and renewing cell populations, while anti-PD-1/PD-L1 drugs do not. However, anti-PD-1/PD-L1 drugs are associated with higher risks of immune-related AEs, including pneumonitis and hypothyroidism, than docetaxel. Nevertheless, in the included studies, these immune-related AEs were efficiently managed with the use of protocol guidelines. In other words, the safety profile of anti-PD-1/PD-L1 therapy was acceptable, suggesting that these drugs might become more popular and widely used in clinical practice.

Some relevant limitations existed in our study. First, to date, only three randomized clinical trials investigating the efficacy and safety of anti-PD-1/PD-L1 antibodies for previously treated NSCLC as second-line treatment have been conducted, so the number of reports incorporated in this meta-analysis was hence limited. However, there were 1141 previously treated patients included in this meta-analysis, and there was little evidence of statistical heterogeneity. As a result, our results can be considered generally reliable. Second, our meta-analysis, like all studies based on aggregated data, did not reach the level of evidence obtainable with a meta-analysis based on individual patient data.

In conclusion, anti-PD-1/PD-L1 therapy may significantly improve the outcomes for patients with pretreated advanced NSCLC, with a favorable safety profile. Therefore, the use of anti-PD-1/PD-L1 therapy in clinical practice is worth further exploring in patients with pretreated advanced NSCLC. More randomized controlled trials with large sample sizes are needed to establish the patient population that would benefit most from this therapy.
